# Electrochemical Detection of Pyocyanin as a Biomarker for *Pseudomonas aeruginosa*: A Focused Review

**DOI:** 10.3390/s20185218

**Published:** 2020-09-13

**Authors:** Fatima AlZahra’a Alatraktchi, Winnie E. Svendsen, Søren Molin

**Affiliations:** 1 Department of Science and Environment, Roskilde University, 4000 Roskilde, Denmark; 2 Department of Biomedicine and Bioengineering, Technical University of Denmark, 2800 Kgs.-Lyngby, Denmark; winnie.svendsen@dtu.dk; 3 The Novo Nordisk Foundation Center for Biosustainability, Technical University of Denmark, 2800 Kgs.-Lyngby, Denmark; sm@bio.dtu.dk

**Keywords:** pyocyanin, electrochemical detection, voltammetry, pseudomonas aeruginosa, diagnosis, infections

## Abstract

*Pseudomonas aeruginosa* (PA) is a pathogen that is recognized for its advanced antibiotic resistance and its association with serious diseases such as ventilator-associated pneumonia and cystic fibrosis. The ability to rapidly detect the presence of pathogenic bacteria in patient samples is crucial for the immediate eradication of the infection. Pyocyanin is one of PA’s virulence factors used to establish infections. Pyocyanin promotes virulence by interfering in numerous cellular functions in host cells due to its redox-activity. Fortunately, the redox-active nature of pyocyanin makes it ideal for detection with simple electrochemical techniques without sample pretreatment or sensor functionalization. The previous decade has seen an increased interest in the electrochemical detection of pyocyanin either as an indicator of the presence of PA in samples or as a tool for quantifying PA virulence. This review provides the first overview of the advances in electrochemical detection of pyocyanin and offers an input regarding the future directions in the field.

## 1. *Pseudomonas aeruginosa* Can Be Identified by a Unique Biomarker

*Pseudomonas aeruginosa* (PA) is on the list of particularly problematic bacterial pathogens published previously by the World Health Organization [1]. PA is an opportunistic pathogen responsible for severe infections in various body systems, including the respiratory tract, the urinary tract, the vascular system and the central nervous system. Furthermore, PA is one of the main causes of nosocomial infections [2]. PA possesses various virulence factors aimed at establishing long-term infections [3,4]. One of the most important PA virulence factors is pyocyanin which is uniquely produced by PA [2]. Pyocyanin has earlier been considered to be a waste product having no actual biological function. However, pyocyanin has, in the past decade, gained wide recognition as one of the crucial factors in the establishment of PA infections [5,6].

Pyocyanin is a redox-active molecule, capable of accepting and donating electrons, which makes it ideal for electrochemical detection [7]. The incorporation of electrochemical sensing into pyocyanin diagnostics offers a variety of advantages, which includes avoiding sample pretreatment, a faster measurement time, increased sample screening yield, lower detection limits and enhanced sensitivity compared to conventional techniques. Moreover, electrochemical sensing provides the opportunity for point-of-care detection of PA unlike conventional techniques that require well-equipped laboratories and trained staff. The most successful modern use of electrochemistry in medicine is the electrochemical sensors for measuring glucose in blood [8]. Pyocyanin has only recently been utilized in miniaturized electrochemical sensing [9]. Intensive research efforts and progress in pyocyanin quantification has therefore only lately been made as a consequence of pyocyanin gaining increasing attention. The electrochemical detection of pyocyanin is therefore a newly emerging field arising from the rapid progression in basic pyocyanin research.

Several reviews have covered the general electrochemical detection of common pathogens, including PA [10,11,12]. However, to date, there has been no review of the numerous attempts of pyocyanin detection, nor of associated clinical and research applications. Here, we provide the first focused overview of the current electrochemical detection of pyocyanin. The review provides a comprehensive description of the different electrochemical approaches utilizing the redox-active nature of pyocyanin. Finally, the review summarizes the applications of electrochemical pyocyanin sensing in clinical and bacterial research settings and points to crucial directions for future research.

## 2. Importance of Pyocyanin as Detection Target

PA is the only bacteria capable of producing the redox-active molecule pyocyanin [13]. Pyocyanin was the name given by Fordos in 1859 due to the blue pigment seen on PA infected wound dressings [14]. Several studies have repeatedly proven the significance of pyocyanin in the virulence and pathogenicity of PA infections [7]. Amongst the virulence characteristics is the antagonistic effects of pyocyanin on host cells that result in cellular damage and death. Pyocyanin has the ability to induce oxidative stress, partially due to its capability of increasing intracellular levels of reactive oxygen species (ROS). It has also been suggested that pyocyanin contributes to biofilm formation by promoting cell-to-cell interactions between PA cells [2,5,6,7,14,15].

### 2.1. Nonelectrochemical Techniques Used for Pyocyanin Detection

Since the discovery of pyocyanin, pyocyanin has been studied in microbial or clinical research through standardized procedures. Pyocyanin is extracted from PA cultures or clinical specimens using chloroform extraction protocols from supernatants of the culture or clinical samples [16,17]. Subsequently, the pyocyanin is quantified by high performance liquid chromatography (HPLC) or spectrophotometry with detection limits in the micromolar range [18,19,20]. Pyocyanin chemistry has also been characterized by Proton Nuclear Magnetic Resonance (Proton NMR) and Fourier Transform Infrared Spectroscopy (FTIR) [21]. Common for these techniques is the dependency on centralized laboratories, time-consuming pyocyanin extraction and purification, the need for trained personnel and inevitable complex workflow. Direct detection of pyocyanin in clinical samples and bacterial cultures without sample pretreatment has been reported possible by Surface Enhanced Raman Spectroscopy (SERS) and Surface Enhanced Resonance Raman Scattering (SERRS) [22,23,24]. Thus, SERS may be a possible candidate for simple pyocyanin detection that also has the potential to be developed into a point-of-care system.

### 2.2. Basic Electrochemistry for Pyocyanin Studies

**Figure 1:**
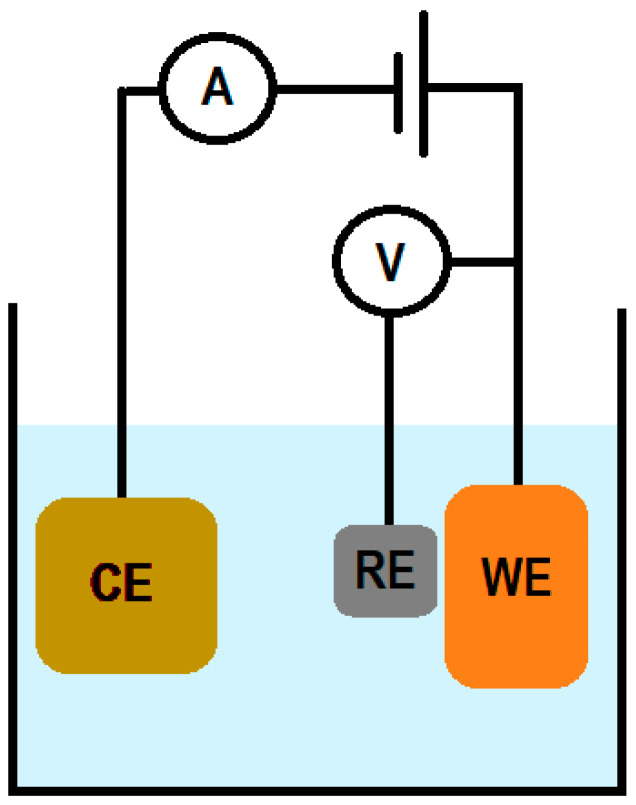
A three-electrode system consists of a working electrode (WE) where the reaction of interest occurs, a counter electrode (CE) to pass the needed current to WE and a reference electrode (RE) which the WE potential is measured against. A: Ammeter. V: Voltmeter.

Electrochemical sensors used for pyocyanin detection usually consists of a three-electrode configuration, where a current moves between a counter electrode (CE) and a working electrode (WE), and the potential of the WE is measured against the reference electrode (RE) (see Figure 1) [25]. The electrochemical detection of pyocyanin generally relies on its capability to diffuse to the proximity of the working electrode surface. Here, the pyocyanin interacts electrochemically with the electrode by oxidation or/and reduction and a perturbation in the current will be measured by the electrochemical measuring equipment, the potentiostat [9].

**Figure 2:**
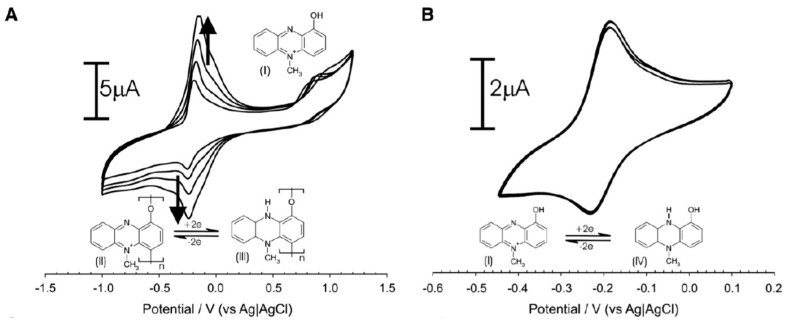
(**A**) Pyocyanin can undergo a reversible redox-reaction involving two electrons and an irreversible phenolic oxidation. (**B**) The phenolic oxidation and polymerization can be excluded by limiting the potential window. Adapted from Sharp et al. 2010 [9].

The electrochemical sensors require no functionalization due to the direct identification of the redox potential of pyocyanin [9]. The electrochemical activity entails a reversible redox-reaction involving two electrons often depicted as negative potentials in voltammograms (Figure 2) [9,26]. Irreversible phenolic oxidation occurs at higher positive potentials, which is responsible for polymerization of pyocyanin (Figure 2A). The polymerization leads to increasing peak heights for the pyocyanin oxidation and reduction with repetitive scan cycles. Polymerization can be avoided by limiting the scan range to the reversible redox reaction window and thereby obtaining exclusive conversion between the soluble, monomeric form of pyocyanin (Figure 2B).

Electrochemical detection allows a fast and simple quantification of pyocyanin at a fair cost [27]. Furthermore, the electrochemical measurements are capable of quantifying pyocyanin to limits of detection and sensitivity beyond conventional approaches [25]. Potentiostats can be bought in various sizes, including handheld, portable devices, making point-of-care use technologically possible.

## 3. The Start of Electrochemical Pyocyanin Detection

The vast majority of electrochemical pyocyanin detection reported earlier relies on voltammetric techniques. The first electrochemical sensing of pyocyanin was reported in 1996, where a hanging mercury drop electrode with a surface area of 1.97 mm^2^ was employed as the WE, a platinum electrode as the CE and Ag/AgCl as the RE [28]. Pyocyanin was detected by adsorptive stripping voltammetry (AdSV) with an oxidation peak at the potential of −0.17 V against Ag/AgCl. The limit of detection (LOD) was 2.0 nM in diluted Mueller–Hinton broth [28].

It was not until more than a decade later that the next approaches for electrochemical detection of pyocyanin appeared. In 2009, cyclic voltammetry (CV), differential pulse voltammetry (DPV) and Faradaic impedance spectroscopy (FIS) were used to measure the generation of pyocyanin associated with the formation or inhibition of biofilm [29]. Additionally, also in 2009, Sharp et al. used a carbon fiber tow as the WE to demonstrate that pyocyanin can be measured in a miniaturized setup [9]. A cyclic voltammetric assessment showed reversible pyocyanin transformations at −0.18 and −0.25 V and a nonreversible oxidation at +0.85 V. The peak at the negative potential was used for quantification of pyocyanin by square wave voltammetry (SWV), achieving an LOD of 30 nM in pH adjusted Britton–Robinson buffer.

In the following years, several pyocyanin detection approaches were published using disposable screen-printed electrochemical electrodes [25,30,31,32]. The sensors were single-used and it was argued that the disposability of the electrodes makes them ideal for irreversible electrochemical measurements such as targeting the phenolic oxidation potential of pyocyanin. In 2016, a proof-of-concept based on a disposable electrochemical setup showed the possible quantification of pyocyanin at positive potentials using cyclic voltammetry [32]. The same group also demonstrated that an LOD of 125 nM could be achieved by amperometric measurements at the positive oxidation potential of pyocyanin in complex media containing various redox-active compounds [25].

Electrode modifications or in-house fabricated sensors were developed to further enhance the sensing performance compared to the earlier reported screen-printed electrodes [26,27,33]. Several sensor designs have been described in the literature, ranging from clean-room fabricated nanograss sensors, conventional rod-electrode setups to cheaper models screen-printed on paper [23,31,32]. The fabrication techniques included shadow-printing, ink-jet printing, atomic layer deposition, electrodeposition and electron-beam deposition, while the electrodes where modified with nanoparticles, carbon-nanotubes and nanoalloys in addition to biological modifications to optimize the sensitivity and detection limit [29,30,33,34,35,36,37,38,39].

Gold and carbon are the most reported common electrode materials used for electrochemical pyocyanin detection. However, studies have shown that carbon constitutes a more suitable material for pyocyanin detection due to the fit between the benzene ring in pyocyanin and the carbon molecular structure. Table 1 shows an overview on studies that have developed pyocyanin sensing (methods or sensors) by utilizing voltammetric techniques. The wide linear detection range and the low LOD values reported in the literature cover physiologically relevant concentrations and enable a precise analysis of microbiological studies of pyocyanin secretion. The variety of experimental setups, sensor fabrication methods and analytical procedures point towards diverse and innovative electrochemical approaches for pyocyanin detection in relevant clinical and microbiological settings. In all reported studies, the voltammetric identification of pyocyanin relied on potential-specific selectivity. Studies have electrochemically addressed the contribution of possible interfering molecules, suggesting that the characteristic potentials, at which pyocyanin is oxidized, lie outside the interference window of the other redox-active molecules [25,32]. Another study thoroughly investigated the ability to electrochemically distinguish pyocyanin when PA is cocultured with other clinically relevant bacteria [40]. It was demonstrated that the cocultured species did not generate redox-active metabolites that interfered with the unique electrochemical signal of pyocyanin. As both the reversible redox reaction and the phenolic oxidation of pyocyanin seemingly have limited to no interference from the background in biological samples, it is possible to use both square wave voltammetry and amperometry for the quantification of pyocyanin. However, there might be a higher risk of detecting currents from unspecific reactions when applying amperometric techniques. On the other hand, cyclic voltammetry may only be useful for proof-of-concept purposes due to the high contribution of nonfaradaic currents and the need for high scan rates for detectable reversible currents of pyocyanin.

## 4. Electrochemical Detection of Pyocyanin

### 4.1. Amplification of Pyocyanin Sensing

To facilitate enhanced electrochemical pyocyanin detection, several approaches have focused on amplifying the electrochemical signal of pyocyanin. Physical modifications of the electrodes by nanostructures such as nanoparticles and carbon nanotubes can enhance the sensitivity and LOD but may reach a barrier where the optimization has reached its limit. Therefore, alternative approaches have been invented to further optimize pyocyanin detection in terms of detection range and LOD. This has been conducted either by biologically inducing more pyocyanin excretion or biochemically amplifying the existing pyocyanin signal. *The biologically assisted amplification* is based on adding chemicals that biologically will stimulate PA to produce pyocyanin. Amino acids are key components for bacterial growth. One study showed that pyocyanin production was upregulated by several types of amino acids (proline, histidine, arginine, leucine, tyrosine, and valine) when added to PA cultures [45]. This is especially useful in settings where the presence of PA in multibacterial cultures is to be detected. Upregulating pyocyanin production can be beneficial for qualitative PA identification when the cell number is low and there is a risk that the sensor will not be capable of detecting small pyocyanin concentrations.

*The biochemical amplification* is mainly useful for the detection of low pyocyanin concentrations where quantification is valuable. Diverse mediators can engage in redox-cycling reactions with a biobased redox capacitor. Redox cycling with catechol–chitosan as a redox capacitor can amplify electrochemical signals for detecting pyocyanin. This method showed that an LOD of 50 nM could be achieved [39]. A similar approach using a bioelectrochemical sensing system based on a whole-cell redox reactivation module was constructed using *Shweanella oneidensis* cells as the bioelectrocatalyst and lactate as the electron donor [38]. This setup could regenerate reductive pyocyanin from its oxidative state and this way enable repeated pyocyanin registration. With this method it was possible to reach an LOD of 47 pM [43].

### 4.2. Simultaneous Profiling of PA Metabolites

Pyocyanin is not the only redox-active compound produced by PA. PA secretes a number of redox-active phenazines and quorum sensing (QS) molecules, including pyoverdine, Pseudomonas Quinolone Signal (PQS), 2-heptyl-4-hydroxyquinoline (HHQ), 1-phenazine-1-carboxamide (PCN), phenazine-1-carboxylic acid (PCA), and 5-methylphenazine-1-carboxylic acid (5-MCA) [46,47]. Although only pyocyanin is exclusively produced by PA, the simultaneous detection of several metabolites is advantageous both for bacterial research purposes, but also for using the presence of the compounds as a molecular signature for PA. Several studies have conducted multiple molecule detection using electrochemical measurements. These studies have mapped the potential values belonging to the aforementioned metabolites and do not overlap, which allows the simultaneous detection of the compounds in one measurement, e.g., through DPV or SWV in the relative potential range of −0.7 to 1.5 V [35,41,48,49,50,51,52].

Although the majority of reported electrochemical detection of pyocyanin employs voltammetric techniques, pyocyanin measurement by electrochemical impedance spectroscopy (EIS) has only once been described in the literature. The study stated that a characteristic impedance signature changed due to the presence of phenazines including pyocyanin [53].

### 4.3. Detection of Pyocyanin from Biofilms on Agar

The detection of pyocyanin excretion from PA cultures on agar plates was studied by a number of routes. Pioneering was Bellin et al. who microfabricated an array of electrodes with an agar layer placed on top of it. This way it was possible to spatially monitor pyocyanin concentration gradients produced across a PA colony. However, it was necessary to grow the bacteria on an agar plate and then transfer it to the electrode array for measurements. The output would be single-time measurements of a “line scan” across the bacterial colony showing the variance of the pyocyanin concentration [41].

The complexity of the fabrication and the handling of this setup gave rise to a simpler approach where different PA cell counts were cultured in an agar plate. An electrochemical screen-printed sensor was placed beneath the agar. The pyocyanin was then measured every hour using SWV, showing an increasing pyocyanin concentration over time. This study aimed at gaining early PA identification on agar plates compared to the conventional visual inspections. Using this approach it was possible to confirm the presence of PA several hours before visual analysis [54]. Although this method was simple and cheap, it was not intended for the spatial monitoring of pyocyanin gradients.

The implications of the direct detection of bacterial pyocyanin responses allow for the investigation of instant bacterial behavior towards different stress factors, such as antibiotics, other microbial species, nutrients, oxygenation, etc. Screen-printed commercial electrodes were used to detect the pyocyanin response when PA biofilms were exposed to increasing concentrations of the antibiotic colistin sulfate. It was demonstrated that pyocyanin production was reduced by 68% and 82% when exposed to 16 and 100 mg/L of the antibiotic, respectively [30].

### 4.4. Scanning Electrochemical Microscopy for Pyocyanin Investigation

Scanning electrochemical microscopy (SECM) is a technique that measures the local electrochemical behavior of substrate interfaces. Spatially resolved electrochemical signals can be acquired by measuring the current at an ultramicroelectrode tip as a function of the tip position scanned over a region of interest. An SECM was utilized to measure the pyocyanin produced in the proximal to a PA biofilm. The study revealed a pyocyanin gradient extending several hundred microns from the biofilm surface [55]. The same group used SECM to investigate pyocyanin as a QS molecule in aggregate populations. By measuring the pyocyanin production of PA grown in microtraps, it was discovered that QS occurs in aggregates as small as 500 cells [56]. Another study demonstrated the simultaneous electrochemical measuring and imaging of pyocyanin and other metabolites in biofilms using an SECM coupled to an electrochemical camera chip. This approach gives a unique resolution of the electrochemical activity of biofilms [50]. It is worth noticing that in the SECM setups, redox mediators have been used to generate a feedback approach curve.

The simultaneous detection of pyocyanin while optically imaging the bacteria was possible using transparent ultramicroelectrode arrays [26]. This could enable the real-time or online detection of pyocyanin during the visualization of the cells. Although the ultramicroelectrode array did not allow for an LOD better than the LODs reported by peer researchers, and the linear detection range was reported to be 1–250 µM, which is a larger range than has been documented earlier.

## 5. Towards Clinical Applications

PA is associated both with severe acute infections—in particular in hospitals and intensive care units (ICUs)—and with persistent hard-to-cure infections in many different types of patients, and successful therapy requires interference when the bacterial populations are small and susceptible to antibiotics. As such, it is highly important to develop efficient diagnostic methods with high sensitivity and rapid signal detection. Despite the intrinsic tolerance to many antibiotics, it is still possible to control and eradicate *P. aeruginosa* populations if discovered very early, before they replicate to form large populations with diverse physiologies and enter into adaptive evolution processes. Very often, *P. aeruginosa* is just one of many different microbial species, and it is therefore possible for the organism to hide in the respective microbiota. This scenario is particularly challenging for a successful diagnosis, and again this challenge can only be met by fast, specific and sensitive tracing methods such as pyocyanin monitoring [57].

Today, PA is clinically diagnosed by sampling relevant body fluids and plating them on PA selective agar plates. After 2–5 days of culturing, the plates are visually inspected and if growth appears similar to the characteristic PA morphology and color, the sample is determined PA positive [58]. The problem of this well-established method is that it is labor intensive, takes several days and is dependent on the initial PA cell number in the sample. If the cell number is too low, it is impossible for the bacteria to grow on the plates. Furthermore, other coexisting microorganisms in the patient sample might overgrow on the plate and thus PA can be overseen [59,60]. These major issues with bacterial culture leads to low sensitivity in PA identification, which has been repeatedly reported in the literature [61,62]. As there is no simple alternative to bacterial culture, clinics widely rely on this technique [60]. A Polymerase Chain Reaction (PCR) of the 16S ribosomal DNA (16S rDNA) can additionally be used as a precise PA diagnostic approach [63]. However, a PCR of the 16S rDNA requires DNA extraction and purification from the patient samples in addition to being dependent on centralized laboratories with trained staff. The detection of pyocyanin as a biomarker for an early diagnosis of PA is therefore a potential alternative to bacterial culture or PCR of PA. The advantages of pyocyanin detection in human fluid samples as an indicator for PA infections are manifold, including the high sensitivity, rapid measurement run time and the potential of point-of-care development.

To successfully apply the electrochemical detection of pyocyanin for PA identification in clinical samples, several studies measured human fluids spiked with pyocyanin [31,32,48]. The investigated human fluids covered urine, blood, bronchial lavage, sputum, saliva and hypertonic saline for laryngeal aspirate suctions as well as simulated wound fluid and artificial sputum medium [25,33,37,42,43]. PA is a urinary tract pathogen, which makes urine an obvious body fluid to develop pyocyanin detection in. Urine is also an interesting fluid for pyocyanin detection as various research has proven that infected patients excrete pyocyanin with urine regardless of the foci of infection [64,65]. Blood is also relevant as a target body fluid for pyocyanin detection as PA bloodstream infections have significant patient mortality [66]. Early detection of PA in blood is therefore important for appropriate initial antimicrobial treatment strategies. Wound fluids are similarly relevant as patients with chronic wounds often suffer from PA infections [43].

Airway secretions, including sputum, bronchial lavage and laryngeal aspirate suctions, are major sampling targets for patients with lung infections, especially cystic fibrosis patients [62]. Given the high incidence of chronic airway colonization by PA in patients with cystic fibrosis, the majority of research on pyocyanin has primarily been focused on its effect on this disease’s progression [5]. Electrochemical measurements with an SWV detection of blood did not show any background peak at the negative potentials where pyocyanin was expected to oxidize. Sputum, saliva, urine and hypertonic saline showed background peaks overlapping with the peak of pyocyanin [31,32]. This was solved by detecting pyocyanin at its positive phenolic oxidation potential where no interference from the background occurs [25,32].

A clinical trial has been performed for the electrochemical detection of PA via pyocyanin in chronic wound samples from patients. The trial included 12 patients with chronic wounds. Wound fluids and biofilm samples were collected for analysis with SWV detection utilizing the negative potential assigned to pyocyanin. Only 7.5 µL of wound exudate was needed for each analysis. The sensing surface was covered with a polymeric membrane to achieve the reduced amount of the sample volume. The electrochemical results were compared against a 16S rRNA profiling of the samples, revealing a sensitivity of 71% and a specificity of 57% for the detection of PA [67].

In another innovative approach, electrodes were printed onto the fingers of a glove to create a point-of-use electrochemical pyocyanin screening tool [44]. The glove-based sensor can be used to swipe a surface for screening of the presence of PA. This approach could especially be useful in out-patient clinics where risk of cross-contamination between patients is high.

## 6. Pyocyanin Detection in Clinical Isolates

In the previous section, we reviewed the attempts of using pyocyanin as an indicator for the presence of PA in human body fluids. Another direct clinically relevant implication of the fast sample screening electrochemical techniques offer, is the quantification of pyocyanin in PA isolates from patients. Isolates are typically collected by plating patient samples on selective plates followed by isolating single PA colonies. Since pyocyanin is a QS regulated molecule reflecting the regulatory and physiological state of *P. aeruginosa*, the use of monitoring methods such as the described electrochemical sensors will have a great impact on the research. The knowledge gained by screening bacterial isolates combined with a prospective study of the patient history can reveal valuable information about bacterial responses to antimicrobial treatment or environmental stress factors [5,68].

Most reported studies have been focusing on proof-of-concept research on wildtype laboratory PA strains such as PAO1 and PA14. An actual electrochemical detection of pyocyanin in clinical isolates was conducted by Sismaet et al. on 94 different isolates from patients with hospital-acquired infections or patients with cystic fibrosis. The results proved that all the isolates produced pyocyanin [42]. This is an important finding for the direct identification of PA in clinical samples, as nonproducing PA isolates would lead to false negatives when using pyocyanin as a biomarker for PA. Thus, it is important that pyocyanin detection for PA diagnostics should primarily be applied in the early stages of infection where PA has not yet lost the pyocyanin related genes (PhzM and PhzS) [5].

Another study electrochemically investigated the impact of other pathogenic microbes on pyocyanin production from PA cultures, finding that both growth media and cocultures significantly influence the pyocyanin production [52]. The complex molecular interactions between secreted compounds, in addition to the microbiological interaction with regard to uptake and the growth media in which the bacteria are cultured, are all factors that are of microbiological interest to investigate to understand PA behavior.

The simplicity of electrochemical sensing allows for the fast and sensitive studying of the dynamics of microbiological conditions affecting pyocyanin production and has implications for basic and clinical understanding of PA. However, when comparing pyocyanin quantification across reported studies, it is important to take into consideration that the pyocyanin diffusion is dependent on the sample matrix. Due to the lack of standardized growth media and standardized media mimicking real biological samples or body fluids, there will be significant variations between reported concentrations.

## 7. Future Directions

Pyocyanin has been demonstrated to impact the cellular function of host systems [2,5,6]. The electrochemical detection of pyocyanin has proven its worth as a potential diagnostic approach for indicating the presence of PA and as a tool for a rapid quantitative screening of the molecule in laboratory settings. The future directions can be summarized in two categories: (1) the technological development of electrochemical sensing and (2) the applications of electrochemical sensing.

*The technological development* of electrochemical sensing of pyocyanin has already provided us with basic tools that can measure pyocyanin with high accuracy and to clinically relevant detection limits. Although the current electrochemical pyocyanin detection development is comprehensive and covers several clinical and microbiological needs, the current literature is predominantly limited to in vitro or ex vivo studies. However, assessing the pyocyanin development from early infection to late chronic infection in vivo can provide insights to the adaptation of PA in patients. The effects of antimicrobials on the production of pyocyanin in vivo might also reveal information about the effectiveness of certain pharmacological agents and hence allow more personalized medication. Implementing electrochemical sensors in mice for the detection of other metabolites has already been reported in the literature and therefore there is no technological hinderance for the detection of pyocyanin in vivo. Another relevant future technological development is the creation of in situ sensors for the electrochemical detection of pyocyanin. In situ sensors would allow an on-the-spot detection of pyocyanin during, e.g., surgeries where samples are difficult to collect. In cystic fibrosis patients, who especially suffer from PA infections, it could be relevant to develop in situ sensors that can be inserted through the nose to the sinus or through the mouth to the lower airways.

The application of electrochemical sensors for clinical investigations can already be executed with no further technological development. The electrochemical sensing of pyocyanin can help us answer biomedical questions that have been difficult to pursue with conventional methods. An early diagnosis of PA during the early colonization or infection stages is the most important application of electrochemical pyocyanin detection. The understanding of PA disease progression through biomarkers such as pyocyanin might create new insights regarding the adaptation of the bacterium during infection establishment and chronic colonization [6,69,70]. The electrochemical tools can also be used for screening purposes when searching for therapies to minimize the toxicity associated with PA infections. In vivo, in situ and ex vivo electrochemical monitoring of pyocyanin and the high throughput analysis of large sample quantities are likely to be the most productive avenues for future research.

## 8. Concluding Remarks

PA is an opportunistic pathogen causing severe infections in immunocompromised patients [71]. Extensive basic and clinical research has proven that PA has several not yet completely uncovered strategies for establishing infection [61,72]. Virulence factors including pyocyanin are crucial in the adaptation process; however, their exact impact remains unclear [3]. As pyocyanin is a redox-active compound uniquely secreted by PA, it has in the recent years been detected by electrochemical sensing techniques. Electrochemical sensing is capable of quantifying pyocyanin to sub nanomolar limits without a sample pretreatment and within seconds of the measuring time, thus allowing a high throughput and accurate analysis. This newly emerging field has led to various research approaches that seek to understand of the pyocyanin role in the process of infection establishment. The studies have demonstrated that the electrochemical sensing of pyocyanin has the potential to be used as a diagnostic tool in clinical settings to identify PA in patient samples and as a research tool for the rapid screening of pyocyanin concentration in bacterial cultures. Future research is required to establish techniques for the in vivo and in situ electrochemical detection of pyocyanin. Novel developments can help understanding the action of pyocyanin in diseases, which is timely as the clinical relevance of pyocyanin is gaining wide recognition.

## Figures and Tables

**Table 1:** Overview of reports on electrochemical methods for the determination of pyocyanin.

WE Material	Electrode Type/Fabrication	Technique	Electrolyte/Sample Matrix	Detection Potential	LOD [µM]	Linear Range [µM]	Ref
Gold	Screen printed	CV	Mixture of pyoverdine, NAD, NADH, NADP, NADPH, phenazine-C12H8N2, and Lysogeny Broth and Human saliva	+0.699 V vs. Ag	2	2–100	[32]
Gold	Screen printed	Amp	Mixture of pyoverdine, NAD, NADH, NADP, NADPH and Lysogeny Broth and Artificial Sputum Medium	+0.82 V vs. Ag	-	0.125–90	[25]
Gold	Integrated circuit sensing platform	SWV	Agar	−0.33 V vs. Ag/AgCl	2.6	-	[40]
Gold coated nanograss	Deep reactive ion etching and E-beam deposition	Coulometry	Hypertonic saline and airway samples from cystic fibrosis patients	+0.533 V vs. Au	0.172	0.313–25	[41]
Gold coated with a catechol-chitosan film	Electrodeposition and grafting redox-active catechols onto a chitosan film	DPV	Lysogeny broth	Approx. −0.25 V vs. Ag/AgCl	0.050	0.050–40	[39]
Transparent carbon ultramicroelectrode arrays with chitosan gold nanoparticles Planar transparent macroelectrodes	Lithography	SWV	Lysogeny broth with sodium phosphate buffer	−0.245 V vs. SCE	1.6 0.75	1–100 0.75–25	[26]
Carbon	Screen printed	SWV	Lysogeny Broth, urine, bronchial lavages, sputum and heparinized blood	Approx. −0.25 V vs. AgCl	0.13–1.81	1–100	[31]
Carbon	Shadow printed	SWV	Lysogeny Broth	−0.55 V vs. Carbon	0.095	1–40	[27]
Carbon	Screen printed	SWV	Trypticase soy broth	−0.25 V vs. Ag/AgCl	0.038	0–100	[42]
Carbon	Screen printed	SWV	Agar-Au/Ag nanoalloy with human serum, whole blood, saliva	−0.3 V vs. Ag/AgCl	0.04	0.12 to 25	[33]
Carbon	Pad printed	SWV	Britton–Robinson, Simulated wound fluid, Human serum	−0.2 V vs. Ag/AgCl	0.15 0.087 0.169	0.336–10 0.336–20 0.183–20	[43]
Carbon	Printed on glove	SWV	Hydrogel	−0.5 V vs. Ag/AgCl	3.33× 10^−3^	0.01–1	[44]
Carbon nanotubes	Ink-jet printed	SWV	Wound fluid simulant	−0.300 V vs. Ag/AgCl	0.1	0.1–100	[37]
Carbon fiber	Tow	SWV	Britton–Robinson Buffer and *P. aeruginosa* Broth	−0.18 V vs. Ag/AgCl	0.030	1–100	[9]
Graphite	Rod	DPV	Lysogeny Broth	−0.270 vs. Ag/AgCl	-	1–71	[29]
Biofilm colonized carbon cloth	*Shewanella oneidensis* cells as the bioelectrocatalyst and lactate as electron donor	CV	Human serum, blood plasma, saliva spiked in mineral medium plus LB and electron donor (glucose/lactate)	−0.41 V vs. SCE	4.7 × 10^−5^	0.0001–0.1	[38]
Boron-doped diamond	Rod	DPV	Acetate buffer, acetonitrile and spiked sputum	−0.15 V Ag/AgCl	0.05	2–100	[35]
Mercury	Hanging drop	Adsorptive stripping voltammetry	Mueller–Hinton broth diluted in ammonia buffer	−0.17 V vs. Ag/AgCl	2.0× 10^−3^	0.002–0.3	[28]

Abbreviations: WE—working electrode, LOD—limit of detection, CV—cyclic voltammetry, SWV—square wave voltammetry, DPV—differential pulse voltammetry SCE—saturated calomel electrode, Ag/AgCl—silver/silver chloride.

## References

[1] Tacconelli E., Carrara E., Savoldi A., Harbarth S., Mendelson M., Monnet D.L., Pulcini C., Kahlmeter G., Kluytmans J., Carmeli Y. (2018). Discovery, research, and development of new antibiotics: The WHO priority list of antibiotic-resistant bacteria and tuberculosis. Lancet Infect. Dis..

[2] Hall S., McDermott C., Anoopkumar-Dukie S., McFarland A.J., Forbes A., Perkins A.V., Davey A.K., Chess-Williams R., Kiefel M.J., Arora D. (2016). Cellular effects of pyocyanin, a secreted virulence factor of Pseudomonas aeruginosa. Toxins.

[3] Strateva T., Mitov I. (2011). Contribution of an arsenal of virulence factors to pathogenesis of Pseudomonas aeruginosa infections. Ann. Microbiol..

[4] Lyczak J.B., Cannon C.L., Pier G.B. (2000). Establishment of Pseudomonas aeruginosa infection: Lessons from a versatile opportunist. Microbes Infect..

[5] Rada B., Leto T.L. (2013). Pyocyanin effects on respiratory epithelium: Relevance in Pseudomonas aeruginosa airway infections. Trends Microbiol..

[6] Lau G.W., Hassett D.J., Ran H., Kong F. (2004). The role of pyocyanin in Pseudomonas aeruginosa infection. Trends Mol. Med..

[7] Dietrich L.E.P., Price-Whelan A., Petersen A., Whiteley M., Newman D.K. (2006). The phenazine pyocyanin is a terminal signalling factor in the quorum sensing network of Pseudomonas aeruginosa. Mol. Microbiol..

[8] Cash K.J., Clark H.A. (2010). Nanosensors and nanomaterials for monitoring glucose in diabetes. Trends Mol. Med..

[9] Sharp D., Gladstone P., Smith R.B., Forsythe S., Davis J. (2010). Approaching intelligent infection diagnostics: Carbon fibre sensor for electrochemical pyocyanin detection. Bioelectrochemistry.

[10] Amiri M., Bezaatpour A., Jafari H., Boukherroub R., Szunerits S. (2018). Electrochemical methodologies for the detection of pathogens. ACS Sens..

[11] McEachern F., Harvey E., Merle G. (2020). Emerging technologies for the electrochemical detection of bacteria. Biotechnol. J..

[12] Kuss S., Amin H.M.A., Compton R.G. (2018). Electrochemical detection of pathogenic bacteria-recent strategies, advances and challenges. Chem. Asian J..

[13] Reyes E.A.P., Bale M.J., Cannon W.H., Matsen J.M. (1981). Identification of Pseudomonas aeruginosa by pyocyanin production on Tech agar. J. Clin. Microbiol..

[14] Turner J.M., Messenger A.J. (1986). Occurence, biochemistry and physiology of phenazine pigment production. Adv. Microb. Physiol..

[15] Jayaseelan S., Ramaswamy D. (2014). Pyocyanin: Production, applications, challenges and new insights. World J. Microbiol. Biotechnol..

[16] King M.M., Guragain M., Sarkisova S.A., Patrauchan M.A. (2016). Pyocyanin extraction and quantitative analysis in swarming pseudomonas aeruginosa. Bio-Protocol.

[17] Frank L., DeMoss R. (1958). On the biosynthesis of pyocyanine. J. Bacteriol..

[18] Watson D., Macdermot J., Wilson R., Cole P.J., Taylor G.W. (1986). Purification and structural analysis of pyocyanin and 1-hydroxyphenazine. Eur. J. Biochem..

[19] Conference I., Sciences M., Penh P. Production, extraction comparison of spectral analysis of pyocyanin diffusible pigment from pseudomonas aeruginosa. Proceedings of the 2nd International Conference on Chemical, Biological and Medical Sciences (ICCBMS’2013).

[20] Wilson R., Sykes D.A., Watson D., Rutman A., Taylor G.W., Cole P.J. (1988). Measurement of pseudomonas aeruginosa phenazine pigments in sputum and assessment of their contribution to sputum sol toxicity for respiratory epithelium. Infect. Immun..

[21] Laxmi M., Bhat S.G. (2016). Characterization of pyocyanin with radical scavenging and antibiofilm properties isolated from Pseudomonas aeruginosa strain BTRY1. 3 Biotech.

[22] Bodelón G., Montes-garcía V., López-puente V., Hill E.H., Hamon C., Sanz-ortiz M.N., Rodal-cedeira S., Costas C., Celiksoy S., Pérez-juste I. (2016). Surface-enhanced resonance Raman scattering. Nat. Mater..

[23] Polisetti S., Baig N.F., Morales-soto N., Shrout J.D., Bohn P.W. (2017). Spatial mapping of pyocyanin in pseudomonas aeruginosa bacterial communities using surface enhanced raman scattering. Appl. Spectrosc..

[24] Wu X., Chen J., Li X. (2014). Culture-free diagnostics of Pseudomonas aeruginosa infection by silver nanorod array based SERS from clinical sputum samples. Nanomed. Nanotechnol. Biol. Med..

[25] Alatraktchi F.A., Johansen H.K., Molin S., Svendsen W.E. (2016). Electrochemical sensing of biomarker for diagnostics of bacteria-specific infections. Nanomedicine.

[26] Elliott J., Simoska O., Karasik S., Shear J.B., Stevenson K.J. (2017). Transparent carbon ultramicroelectrode arrays for the electrochemical detection of a bacterial warfare toxin, pyocyanin. Anal. Chem..

[27] Alatraktchi F.A., Noori J.S., Tanev G.P., Mortensen J., Dimaki M., Johansen H.K., Madsen J., Molin S., Svendsen W.E. (2018). Paper-based sensors for rapid detection of virulence factor produced by Pseudomonas aeruginosa. PLoS ONE.

[28] Vukomanovic D.V., Brien J.F., van Loon G.W., Zoutman D.E., Marks G.S., Nakatsu K. (1996). Analysis of pyocyanin from pseudomonas aeruginosa by adsorptive stripping voltammetry. J. Pharmacol. Toxicol. Methods.

[29] Bukelman O., Amara N., Mashiach R., Krief P., Meijler M.M., Alfonta L. (2009). Electrochemical analysis of quorum sensing inhibition. Chem. Commun..

[30] Webster T.A., Sismaet H.J., Chan I.J., Goluch E.D. (2015). Electrochemically monitoring the antibiotic susceptibility of *Pseudomonas aeruginosa* biofilms. Analyst.

[31] Webster T.A., Sismaet H.J., Conte J.L., Chan I., Ping J., Goluch E.D. (2014). Electrochemical detection of Pseudomonas aeruginosa in human fluid samples via pyocyanin. Biosens. Bioelectron..

[32] Alatraktchi F.A., Andersen S.B., Johansen H.K., Molin S., Svendsen W.E. (2016). Fast selective detection of pyocyanin using cyclic voltammetry. Sensors.

[33] Cernat A., Canciu A., Tertis M., Graur F., Cristea C. (2019). Synergic action of thermosensitive hydrogel and Au/Ag nanoalloy for sensitive and selective detection of pyocyanin. Anal. Bioanal. Chem..

[34] Alatraktchi F.A., Dimaki M., Støvring N., Johansen H.K., Molin S., Svendsen W.E. (2020). Nanograss sensor for selective detection of Pseudomonas aeruginosa by pyocyanin identification in airway samples. Anal. Biochem..

[35] Buzid A., Shang F., Reen F.J., Muimhneacháin E., Clarke S.L., Zhou L., Luong J.H.T., O’Gara F., McGlacken G.P., Glennon J.D. (2016). Molecular signature of pseudomonas aeruginosa with simultaneous nanomolar detection of quorum sensing signaling molecules at a boron-doped diamond electrode. Sci. Rep..

[36] Elkhawaga A.A., Khalifa M.M., El-badawy O., Hassan M.A., El-Said W.A. (2019). Rapid and highly sensitive detection of pyocyanin biomarker in different Pseudomonas aeruginosa infections using gold nanoparticles modified sensor. PLoS ONE.

[37] Jarošová R., Mcclure S.E., Gajda M., Jović M., Girault H.H., Lesch A., Maiden M., Waters C., Swain G.M. (2019). Inkjet-printed carbon nanotube electrodes for measuring pyocyanin and uric acid in a wound fluid simulant and culture media. Anal. Chem..

[38] Yang Y., Yu Y.Y., Wang Y.Z., Zhang C.L., Wang J.X., Fang Z., Lv H., Zhong J.J., Yong Y.C. (2017). Amplification of electrochemical signal by a whole-cell redox reactivation module for ultrasensitive detection of pyocyanin. Biosens. Bioelectron..

[39] Kim E., Gordonov T., Bentley W.E., Payne G.F. (2013). Amplified and in situ detection of redox-active metabolite using a biobased redox capacitor. Anal. Chem..

[40] Santiveri C.R., Sismaet H.J., Kimani M., Goluch E.D. (2018). Electrochemical detection of pseudomonas aeruginosa in polymicrobial environments. ChemistrySelect.

[41] Bellin D.L., Sakhtah H., Rosenstein J.K., Levine P.M., Thimot J., Emmett K., Dietrich L.E.P., Shepard K.L. (2014). Integrated circuit-based electrochemical sensor for spatially resolved detection of redox-active metabolites in biofilms. Nat. Commun..

[42] Sismaet H.J., Pinto A.J., Goluch E.D. (2017). Electrochemical sensors for identifying pyocyanin production in clinical Pseudomonas aeruginosa isolates. Biosens. Bioelectron..

[43] Burkitt R., Sharp D. (2017). Submicromolar quantification of pyocyanin in complex biological fluids using pad-printed carbon electrodes. Electrochem. Commun..

[44] Ciui B., Tertiş M., Cernat A., Sǎndulescu R., Wang J., Cristea C. (2018). Finger-based printed sensors integrated on a glove for on-site screening of pseudomonas aeruginosa virulence factors. Anal. Chem..

[45] Sismaet H.J., Webster T.A., Goluch E.D. (2014). Up-regulating pyocyanin production by amino acid addition for early electrochemical identification of Pseudomonas aeruginosa. Analyst.

[46] Chincholkar S., Thomashow L. (2013). Microbial Phenazines.

[47] Mavrodi D.V., Peever T.L., Mavrodi O.V., Parejko J.A., Raaijmakers J.M., Lemanceau P., Mazurier S., Heide L., Blankenfeldt W., Weller D.M. (2010). Diversity and evolution of the phenazine biosynthesis pathway. Appl. Environ. Microbiol..

[48] Buzid A., Reen F.J., Langsi V.K., Muimhneacháin E., O’Gara F., McGlacken G.P., Luong J.H.T., Glennon J.D. (2017). Direct and rapid electrochemical detection of pseudomonas aeruginosa quorum signaling molecules in bacterial cultures and cystic fibrosis sputum samples through cationic surfactant-assisted membrane disruption. ChemElectroChem.

[49] Seviour T., Doyle L.E., Lauw S.J.L., Hinks J., Rice S.A., Nesatyy V.J., Webster R.D., Kjelleberg S., Marsili E. (2015). Voltammetric profiling of redox-active metabolites expressed by Pseudomonas aeruginosa for diagnostic purposes. Chem. Commun..

[50] Bellin D.L., Sakhtah H., Zhang Y., Price-Whelan A., Dietrich L.E.P., Shepard K.L. (2016). Electrochemical camera chip for simultaneous imaging of multiple metabolites in biofilms. Nat. Commun..

[51] Simoska O., Sans M., Fitzpatrick M.D., Crittenden C.M., Eberlin L.S., Shear J.B., Stevenson K.J. (2019). Real-time electrochemical detection of pseudomonas aeruginosa phenazine metabolites using transparent carbon ultramicroelectrode arrays. ACS Sens..

[52] Simoska O., Sans M., Eberlin L.S., Shear J.B., Stevenson K.J. (2019). Electrochemical monitoring of the impact of polymicrobial infections on Pseudomonas aeruginosa and growth dependent medium. Biosens. Bioelectron..

[53] Ward A.C., Connolly P., Tucker N.P. (2014). Pseudomonas aeruginosacan be detected in a polymicrobial competition model using impedance spectroscopy with a novel biosensor. PLoS ONE.

[54] Webster T.A., Sismaet H.J., Sattler A.F., Goluch E.D. (2015). Improved monitoring of *P. aeruginosa* on agar plates. Anal. Methods.

[55] Koley D., Ramsey M.M., Bard A.J., Whiteley M. (2011). Discovery of a bio fi lm electrocline using real-time 3D metabolite analysis. PNAS.

[56] Connell J.L., Kim J., Shear J.B., Bard A.J., Whiteley M. (2014). Real-time monitoring of quorum sensing in 3D-printed bacterial aggregates using scanning electrochemical microscopy. Proc. Natl. Acad. Sci. USA.

[57] Stuart B., Lin J.H., Mogayzel P.J. (2010). Early eradication of pseudomonas aeruginosa in patients with cystic fibrosis. Paediatr. Respir. Rev..

[58] Johansen H.K., Moskowitz S.M., Ciofu O., Pressler T., Høiby N. (2008). Spread of colistin resistant non-mucoid Pseudomonas aeruginosa among chronically infected Danish cystic fibrosis patients. J. Cyst. Fibros..

[59] Xu J., Moore J., Murphy P., Millar B., Elborn J. (2004). Early detection of Pseudomonas aeruginosa—comparison of conventional versus molecular (PCR) detection directly from adult patients with cystic fibrosis (CF). Ann. Clin. Microbiol. Antimicrob..

[60] Dowd S.E., Wolcott R.D., Sun Y., McKeehan T., Smith E., Rhoads D. (2008). Polymicrobial nature of chronic diabetic foot ulcer biofilm infections determined using bacterial tag encoded FLX amplicon pyrosequencing (bTEFAP). PLoS ONE.

[61] Héry-Arnaud G., Nowak E., Caillon J., David V., Dirou A., Revert K., Munck M.R., Frachon I., Haloun A., Horeau-Langlard D. (2017). Evaluation of quantitative PCR for early diagnosis of Pseudomonas aeruginosa infection in cystic fibrosis: A prospective cohort study. Clin. Microbiol. Infect..

[62] Rosenfeld M., Ramsey B.W., Gibson R.L. (2003). Pseudomonas acquisition in young patients with cystic fibrosis: Pathophysiology, diagnosis, and management. Curr. Opin. Pulm. Med..

[63] Spilker T., Coenye T., Vandamme P., Lipuma J.J. (2004). PCR-Based Assay for differentiation of pseudomonas aeruginosa from other pseudomonas species recovered from cystic fibrosis patients. J. Clin. Microbiol..

[64] El-Fouly M.Z., Sharaf A.M., Shahin A.A.M., El-Bialy H.A., Omara A.M.A. (2015). Biosynthesis of pyocyanin pigment by Pseudomonas aeruginosa. J. Radiat. Res. Appl. Sci..

[65] Al-Ani F.Y., Al-Shibib A.S., Khammas K.M., Taher R. (1986). Pyocyanin preparation from Pseudomonas aeruginosa isolated from heterogeneous clinical materials. Folia Microbiol. (Praha).

[66] Micek S.T., Lloyd A.E., Ritchie D.J., Reichley R.M., Fraser V.J., Kollef M.H. (2005). Pseudomonas aeruginosa bloodstream infection: Importance of appropriate initial antimicrobial treatment. Am. Soc. Microbiol..

[67] Sismaet H.J., Banerjee A., McNish S., Choi Y., Torralba M., Lucas S., Chan A., Shanmugam V.K., Goluch E.D. (2016). Electrochemical detection of Pseudomonas in wound exudate samples from patients with chronic wounds. Wound Repair Regen..

[68] Da Silva Filho L.V.R.F., Ferreira F.d.A., Reis F.J.C., De Britto M.C.A., Levy C.E., Clark O., Ribeiro J.D. (2013). Pseudomonas aeruginosa infection in patients with cystic fibrosis: Scientific evidence regarding clinical impact, diagnosis, and treatment. J. Bras. Pneumol..

[69] Murray T.S., Egan M., Kazmierczak B.I. (2007). Pseudomonas aeruginosa chronic colonization in cystic fibrosis patients. Curr. Opin. Pediatr..

[70] Carlsson M., Shukla S., Petersson A.C., Segelmark M., Hellmark T. (2011). Pseudomonas aeruginosa in cystic fibrosis: Pyocyanin negative strains are associated with BPI-ANCA and progressive lung disease. J. Cyst. Fibros..

[71] Koch C., Høiby N. (2000). Diagnosis and treatment of cystic fibrosis. Respiration.

[72] Folkesson A., Jelsbak L., Yang L., Johansen H.K., Ciofu O., Høiby N., Molin S. (2012). Adaptation of Pseudomonas aeruginosa to the cystic fibrosis airway: An evolutionary perspective. Nat. Rev. Microbiol..

